# Flavokawain B Weakens Gastric Cancer Progression via the TGF-*β*1/SMAD4 Pathway and Attenuates M2 Macrophage Polarization

**DOI:** 10.1155/2022/4903333

**Published:** 2022-07-16

**Authors:** Yongzhao Zhu, Weining Fan, Yuanzhen Wang, Huan Ding, Shaoqi Yang, Fang He

**Affiliations:** General Hospital of Ningxia Medical University, Yinchuan, 750004 Ningxia, China

## Abstract

This study was designed to observe the treatment effects of flavokawain B (FKB) on gastric cancer both in SGC-7901 cells and nude mice. When SGC-7901 cells were exposed to 10 *μ*g/mL FKB, cellular proliferative and apoptotic capacities and cell cycle were detected utilizing CCK-8 and flow cytometry assays. The results showed that FKB treatment induced cell apoptosis and G2/M arrest and suppressed cell proliferation for SGC-7901 cells. Western blot results showed that FKB upregulated proapoptotic proteins as well as downregulated antiapoptotic and cell cycle-related proteins in SGC-7901 cells. SMAD4, TGF-*β*1, and TSPAN12 proteins were tested in FKB-induced SGC-7901 cells. Following exposure to FKB, SMAD4, TGF-*β*1, and TSPAN12 expression was augmented in SGC-7901 cells. si-SMAD4 transfection weakened cell apoptosis and accelerated cell proliferation. Furthermore, FKB reversed the change in apoptotic and cell cycle-related proteins induced by si-SMAD4. A nude mouse tumorigenesis model was constructed, which was treated by FKB. In the nude mouse tumorigenesis model, FKB activated the TSPAN12 expression and TGF-*β*1/SMAD4 pathway. Also, FKB treatment prolonged the survival time of nude mice and lowered tumor weight. iNOS and CD86 expression was significantly enhanced, and Arg-1 and CD206 expression was significantly decreased in THP-1 cells cultured in conditioned media from FKB-treated SGC-7901 cells. Additionally, FKB-treated SGC-7901 cells weakened macrophage migration. Collectively, this evidence suggested that FKB accelerated apoptosis and suppressed the proliferation of gastric cancer cells and attenuated M2 macrophage polarization, thereby exerting an anticancer effect on gastric cancer.

## 1. Introduction

Gastric cancer is a gastrointestinal malignant tumor with the highest incidence and fatality rate worldwide [1–3]. Surgical removal is the first choice for gastric cancer treatment [4, 5]. Most patients are already at an advanced stage and have distant metastases at the time of diagnosis [6]. The prognosis remains pessimistic, and 5-year survival rate is less than 20%. However, chemotherapy drugs usually cause severe side effects, such as liver toxicity [7], nephrotoxicity [8], and immunosuppression [9]. Therefore, the development and research of natural product anticancer drugs with low toxicity and high selectivity have become a research hotspot.

The occurrence of gastric cancer is in close relationship with the imbalance of apoptosis and proliferation [10, 11]. Previous research has found that Chinese medicine monomer can lead to cell cycle arrest at different stages, such as Cannabidiol [12], ethanolic extract of *Cordyceps cicadae* [13], and Phloretin [14]. Therefore, regulation of proliferation and apoptosis of cancer cells is a critical direction for gastric cancer therapy. Mammalian cell cycle progression can be mediated by various enzymes. It has been confirmed that cyclin-dependent protein kinase- (CDK-) cyclin complexes are activated in time of the cell cycle, which can be induced and mediated via environmental factors (ultraviolet rays, ionizing radiation, thermal damage, industrial chemicals, etc.) [15–17]. Apoptosis is the prime type of programmed cell death. During the process, cysteine-aspartic protease (caspase) family membranes are activated [18–20]. Therefore, the application of chemical and biological reagents to induce cell cycle arrest, reduce cell proliferation, and even induce cell apoptosis has been become a promising intervention for the therapy of gastric cancer.

Transforming growth factor- (TGF-) *β* is a polypeptide cytokine with multiple biological activities, which is one of the most important tumor suppressor pathways [21, 22]. Abnormal TGF-*β*/SMAD4 pathway contributes to tumorigenesis of gastric cancer [23]. Tetraspanin-12 belongs to the family of four transmembrane proteins, characterized by four transmembrane domains and two extracellular loops [24]. TSPAN12 has been found to participate to various cancers such as ovarian cancer [25], lung cancer [26], and colorectal cancer [27]. However, its expression and role in gastric cancer remain unclear. Flavokawain B (FKB) extracted from the rhizomes of *Alpinia pricei Hayata*, has a strong antiproliferative effect on a variety of cancer cells glioma [28], prostate cancer [29], breast cancer [30], thyroid cancer [31], etc. FKB can exert an antitumor biological activity through multiple mechanisms. For example, FKB could inhibit proliferative capacity and motivate cell cycle arrest in colon cancer cells [32]. Furthermore, FKB suppresses ROS-mediated apoptosis and autophagic cell death of lung adenocarcinoma cells [33]. However, the specific effect of FKB on gastric cancer remains unknown. Furthermore, whether FKB is related to the TGF-*β*/SMAD4 pathway is unclear.

Macrophages exert key roles in the initiation and progression of human cancers [34–36]. Tumor-associated macrophages exhibit enhanced plasticity as well as predominantly show as M2 phenotype, that is, in relation to cancer metastases and undesirable prognosis [37]. Thus, alleviating M2 macrophage polarization within the tumor microenvironment represents a promising therapeutic regimen against gastric cancer. Limited evidence indicates that both FKB and TGF-*β*1-SMAD4 signaling pathway mediate macrophage differentiation. For instance, FKB reduces LPS-triggered proinflammatory IL-6 secretion in macrophages [38]. Moreover, SMAD4 variant facilitates macrophage recruitment as well as inflammatory responses through activating TGF-*β* signaling in thoracic aortic aneurysm and dissection [39]. Herein, we hypothesized that FKB could expedite cell cycle arrest and apoptosis of gastric cancer cells via the TGF-*β*1-SMAD4 signaling pathway as well as weaken the induction capacity of SGC-7901 cells in macrophage polarization.

## 2. Materials and Methods

### 2.1. Cell Culture

Human gastric cancer cell line SGC-7901 was obtained from Chinese Academy of Sciences (Shanghai, China), which was grown in RPMI-1640 medium (Invitrogen, USA) plus 10% fetal bovine serum (FBS), penicillin (100 U/mL) and streptomycin (100 *μ*g/mL). The cells were fostered in a humidified incubator containing 5% CO_2_ at 37°C.

### 2.2. Cell Transfection and Drug Treatment

SGC-7901 cells were seeded in a 6-well plate (2.5 × 10^5^ cells/well) for 24 h. si-SMAD4 (sense: 5′-GCCACUGUAUCCAGCAGAUTT-3′; antisense: 5′-GCCACUGUAUCCAGCAGAUTT-3′) or negative control (NC) synthesized by RIBOBIO (Guangzhou, China) was transfected into cells via Lipofectamine 2000 (Applied Biosystems, Life technologies, USA). Following 48 h of transfection, the cells were harvested.

FKB solution (10 *μ*g/mL; LKT Laboratories, Inc., St. Paul, MN, USA) was prepared in DMSO [33]. FKB was diluted to the concentration of 0.1% using DMSO. Then, SGC-7901 cells were exposed to FKB solution.

### 2.3. Cell Counting Kit-8 (CCK-8) Assay

24 h after treatment with FKB (10 *μ*g/mL) or si-SMAD4, SGC-7901 cells (5.0 × 10^3^) were plated into a 96-well plate. After culturing for 1, 2, 3, and 4 days, the original culture medium was removed, and serum-free medium containing 10 *μ*L CCK-8 solution (Dojindo, Japan) was added to each well and incubated for 1 h at 37°C. Using an enzyme-linked immunoassay instrument, the corresponding absorbance (OD value) was monitored at a wavelength of 570 nm.

### 2.4. Annexin V-FITC/PI Assay

Cells in the logarithmic growth phase were taken and digested utilizing trypsin without EDTA. Then, they were centrifuged to the cell suspension at 2000 rpm for 5 min. The cell pellet was collected, resuspended in PBS buffer, and centrifuged at 2,000 rpm for 5 min. The cells were resuspended with 500 *μ*L binding solution, which were then incubated with 10 *μ*L Annexin V-FITC staining solution (Meilunbio, Dalian, China). Afterwards, 10 *μ*L propidium iodide (PI; Meilunbio) staining solution was added and incubated for 15 min at room temperature in the dark. Within 1 h, apoptotic rate was assessed utilizing flow cytometry (BD Biosciences, USA).

### 2.5. Western Blot

Tissues or cells were fully lysed through RIPA lysis buffer (Beyotime, China). After standing on ice for 30 min, the samples were centrifuged at 13,000 g for 10 min. The supernatant was stored at -20°C. The Bradford method was utilized to determine the protein concentration. After being separated, the sample was transferred to the PVDF membrane (Millipore, USA), followed by being sealed in 5% skimmed milk powder for 2 h. Following being washed with TBST, the diluted primary antibodies were added to the membrane and shook it overnight at 4°C. After being washed again, corresponding HRP-labeled secondary antibody (Santa Cruz, USA) was added dropwise to the membrane and fostered at room temperature for 90 min. The membrane was incubated with ECL luminous liquid (Beyotime). The gel imaging analyzer was used to analyze the gray value. Primary antibodies included TGF-*β*1 (Abcam, USA, ab92486, 1 : 200), SMAD4 (Abcam, ab40759, 1 : 3000), TSPAN12 (Boster, A05472, 1 : 500), caspase-3 (Abcam, ab13847, 1 : 500), caspase-7 (Abcam, ab69540, 1 : 1000), caspase-8 (Abcam, ab25901, 1 : 1000), caspase-9 (Abcam, ab25758, 1 : 300), Bcl-2 (Abcam, ab692, 1 : 500), Bax (Abcam, ab32503, 1 : 5000), cyclin A (Boster, A03889-1, 1 : 1000), cyclin B1 (Boster, BA3168-2, 1 : 200), Cdc2 (Boster, PB0561, 1; 500), Cdc25C (Boster, A01343, 1 : 500), iNOS (Abcam, ab178945, 1 : 1000), CD86 (Abcam, ab239075, 1 : 1000), Arg-1 (Abcam, ab183333, 1 : 1000), CD206 (Abcam, ab64693), CCR2 (Abcam, ab203128, 1 : 1000), and GAPDH (Abcam, ab199553, 1 : 5000) antibodies.

### 2.6. Cell Cycle Analysis

Cells were harvested as well as rinsed by cold PBS twice. Thereafter, they were fixed with cold 70% ethanol at 4°C overnight, followed by treatment with 10 *μ*g/mL RNase at 37°C for 30 min. Afterwards, they were dyed utilizing 50 *μ*g/mL PI for 5 min away from light. Cell cycle was analyzed with flow cytometry (BD Biosciences, USA).

### 2.7. Tissue Specimens

Totally, 20 cases of tumor tissues and normal tissues were collected from 20 gastric cancer patients at the General Hospital of Ningxia Medical University between January 2017 and July 2019. The selection criteria of the samples were as follows: patients with primary gastric cancer who had been pathologically diagnosed as gastric adenocarcinoma; no history of chronic diseases such as cardiovascular and cerebrovascular diseases; no history of other malignant tumors; patients who did not receive any treatment such as radiotherapy or chemotherapy; and patients who had the complete clinical data. All of them provided written informed consent, and the study was approved by the Ethics Committee of General Hospital of Ningxia Medical University (2017-030).

### 2.8. Animals

Female BALB/c nude mice aged 4-6 weeks were purchased from Beijing Laboratory Animal Research Center (China). All animals were reared in an environment of 23 ± 2°C temperature, 55 ± 5% humidity, and 12 : 12 h light/dark cycle. This assay was conducted in line with the guidelines of the General Hospital of Ningxia Medical University Animal Ethics Research Board (2017-030).

### 2.9. Tumor Cell Inoculation

SGC-7901 cells (5 × 10^6^) that were mixed in 200 *μ*L matrix gel were subcutaneously injected into the armpit of the nude mice. After 7 days, these animals were randomly divided into control group (0.1% DMSO) and FKB group. In the FKB group, mice were intraperitoneally injected by 1.5 mg/kg FKB every second day. Meanwhile, control mice were intraperitoneally injected by equal amount of saline. All animals were allowed to survive until they died of natural causes. The tumor tissue was stored at -80°C for protein extraction.

### 2.10. Immunohistochemistry

The tumor tissues from nude mice were formalin-fixed and paraffin-embedded. The sections were treated with xylene and a series of ethanol. Following antigen retrieval, the sections were blocked and stained with primary antibodies against TGF-*β*1 (Abcam, USA, ab92486, 1 : 3000), SMAD4 (Abcam, ab40759, 1 : 3000), and TSPAN12 (Boster, A05472, 1 : 3000), followed by being incubated with secondary antibodies. Then, the sections were counterstained by hematoxylin. The images were acquired under a microscope (Nikon, Japan).

### 2.11. Preparation of Conditioned Media from SGC-7901 Cells

SGC-7901 cells were seeded with 2 × 10^4^ cells/cm^2^ lasting 72 h. After reaching 90% confluence, the cells were administrated with or without 10 *μ*g/mL FKB for 6 h. The medium was exchanged with serum-free fresh medium lasting 24 h. Thereafter, the cells were centrifuged at 2,000 g at 4°C lasting 10 min and were filtered with 0.22 mm filters. The conditioned media were stored at –80°C.

### 2.12. Transwell Assay

THP-1 cells were planted into 6-well plates (2 × 105 cells/well), followed by incubation with conditioned medium. At 48 h, THP-1 cells were harvested for transwell assays. THP-1 cells were maintained in the upper Transwell chamber with FBS-free RPMI-1640 media, and 600 *μ*L RPMI-1640 media plus 20% FBS was added to the lower chamber. After incubation for 24 h, the cells in the lower chamber were fixed with 4% paraformaldehyde as well as stained crystal violet staining solution. Three random fields were chosen.

### 2.13. Statistical Analysis

The GraphPad Prism 7.0 software was utilized to present statistical analyses. Measurement data were denoted as the mean ± standard deviation. Comparisons of two groups were presented via Student's *t* test. More than 2 independent groups were compared by use of one-way analysis of variance. *P* < 0.05 was set as the cutoff value.

## 3. Results

### 3.1. FKB Suppresses Growth and Induces Apoptosis of Gastric Cancer Cells

**Figure 1:**
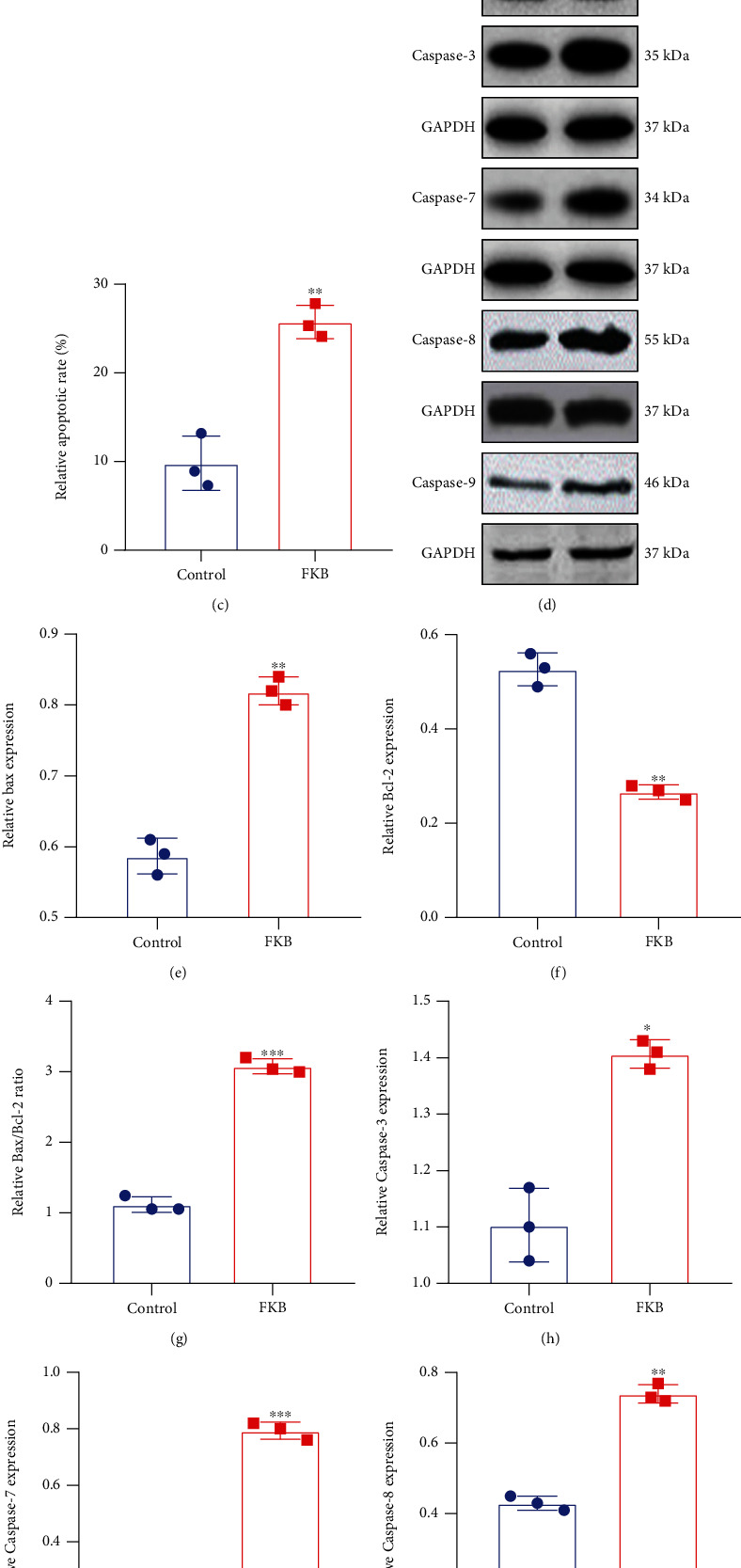
FKB suppresses growth and induces apoptosis of gastric cancer cells. (a) Cell growth curve for SGC-7901 cells after treatment with FKB. (b, c) Apoptosis of SGC-7901 cells was investigated following FKB treatment utilizing Annexin V-FITC/PI test. (d) Representative images of western blots. (e) Bax, (f) Bcl-2, (g) Bax/Bcl-2, (h) caspase-3, (i) caspase-7, (j) caspase-8, and (k) caspase-9 expression was quantified in SGC-7901 cells of the two groups. ^∗^*p* < 0.05; ^∗∗^*p* < 0.01; ^∗∗∗^*p* < 0.001; ^∗∗∗∗^*p* < 0.0001.

The CCK-8 was utilized to detect cell proliferative activity. The results suggested that, compared with controls, FKB treatment slowed down the growth of gastric cancer SGC-7901 cells, which started from day two (Figure 1(a)). Annexin V-FITC/PI test was presented to examine cell apoptosis. In comparison with controls, the apoptotic rate of SGC-7901 cells in the FKB treatment group was significantly increased (Figures 1(b) and 1(c)). Proapoptotic and antiapoptotic proteins were tested in SGC-7901 cells under FKB exposure by western blot (Figure 1(d)). FKB treatment increased Bax expression (Figure 1(e)) and decreased Bcl-2 expression (Figure 1(f)) in SGC-7901 cells. Furthermore, the Bax/Bcl − 2 ratio was distinctly increased following FKB treatment (Figure 1(g)). We also examined Caspase family members in the two groups. FKB treatment increased expression of caspase-3, caspase-7, caspase-8, and caspase-9 (Figures 1(h)–1(k)). Collectively, FKB could activate both extrinsic and intrinsic apoptotic pathways, thereby exhibiting apoptotic effects in gastric cancer cells.

### 3.2. FKB Downregulates Cell Cycle-Related Proteins in Gastric Cancer Cells

**Figure 2:**
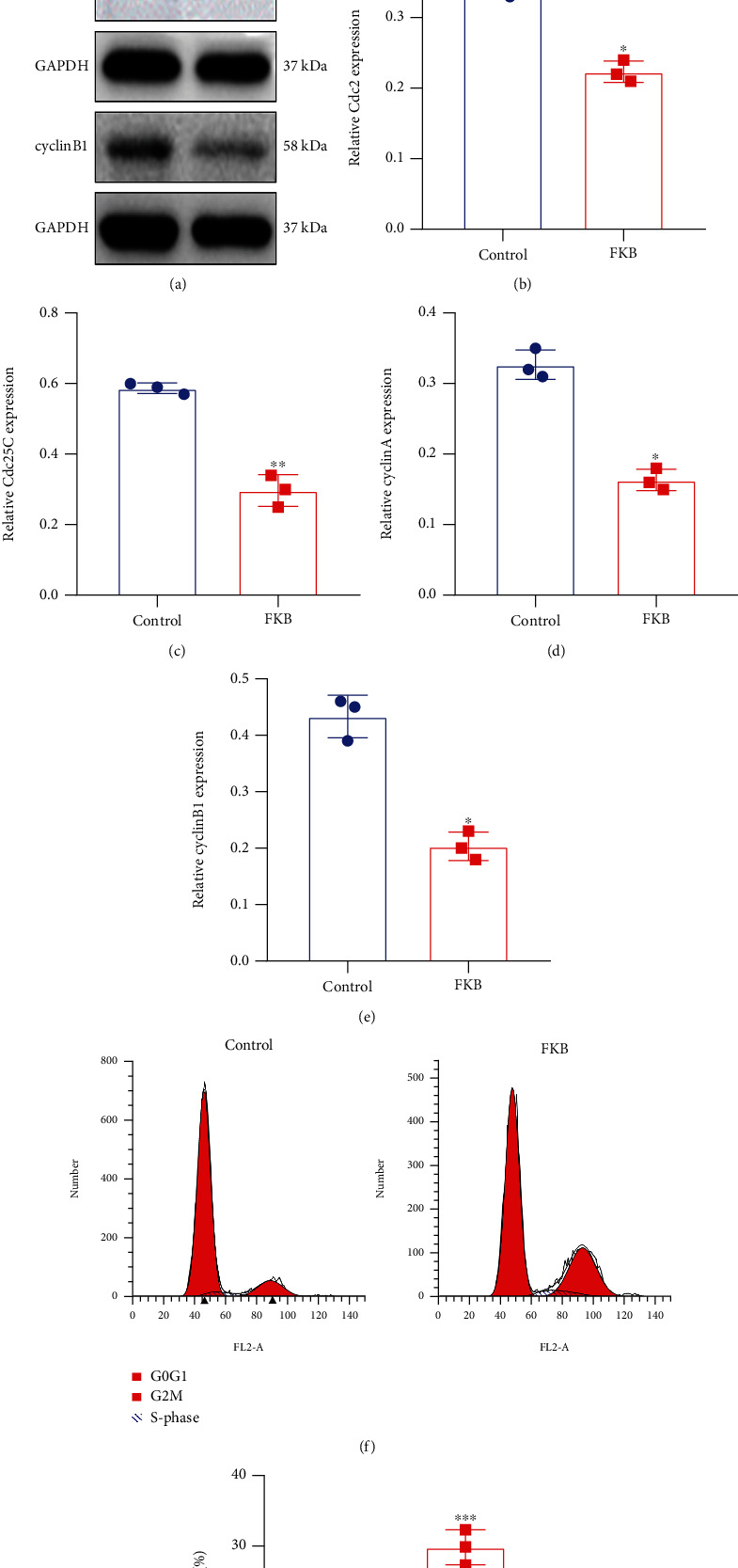
FKB downregulates the expression of cell cycle-related proteins in gastric cancer cells. (a) Representative images of western blots. (b) Cdc2, (c) Cdc25C, (d) cyclin A, and (e) cyclin B1 expression was quantified in SGC-7901 cells of the two groups. (f, g) The flow cytometry analysis of the cell cycle in SGC-7901 cells of the two groups. ^∗^*p* < 0.05; ^∗∗^*p* < 0.01; ^∗∗∗^*p* < 0.001.

We further observed the effects of FKB treatment on cyclins and CDKs during cell cycle progression in SGC-7901 cells (Figure 2(a)). After treatment of 10 *μ*g/mL FKB for 24 h, Cdc2 (Figure 2(b)), Cdc25C (Figure 2(c)), cyclin A (Figure 2(d)), and cyclin B1 (Figure 2(e)) expression was remarkedly reduced in SGC-7901 cells. The flow cytometry analysis of the cell cycle showed that the percentage of G2/M was higher in 10 *μ*g/mL FKB-treated SGC-7901 cells than controls (Figures 2(f) and 2(g)), indicating that FKB triggered G2/M cell cycle arrest in gastric cancer cells.

### 3.3. Inactivation of the TSPAN12 Expression and TGF-*β*1/SMAD4 Pathway in Gastric Cancer

**Figure 3:**
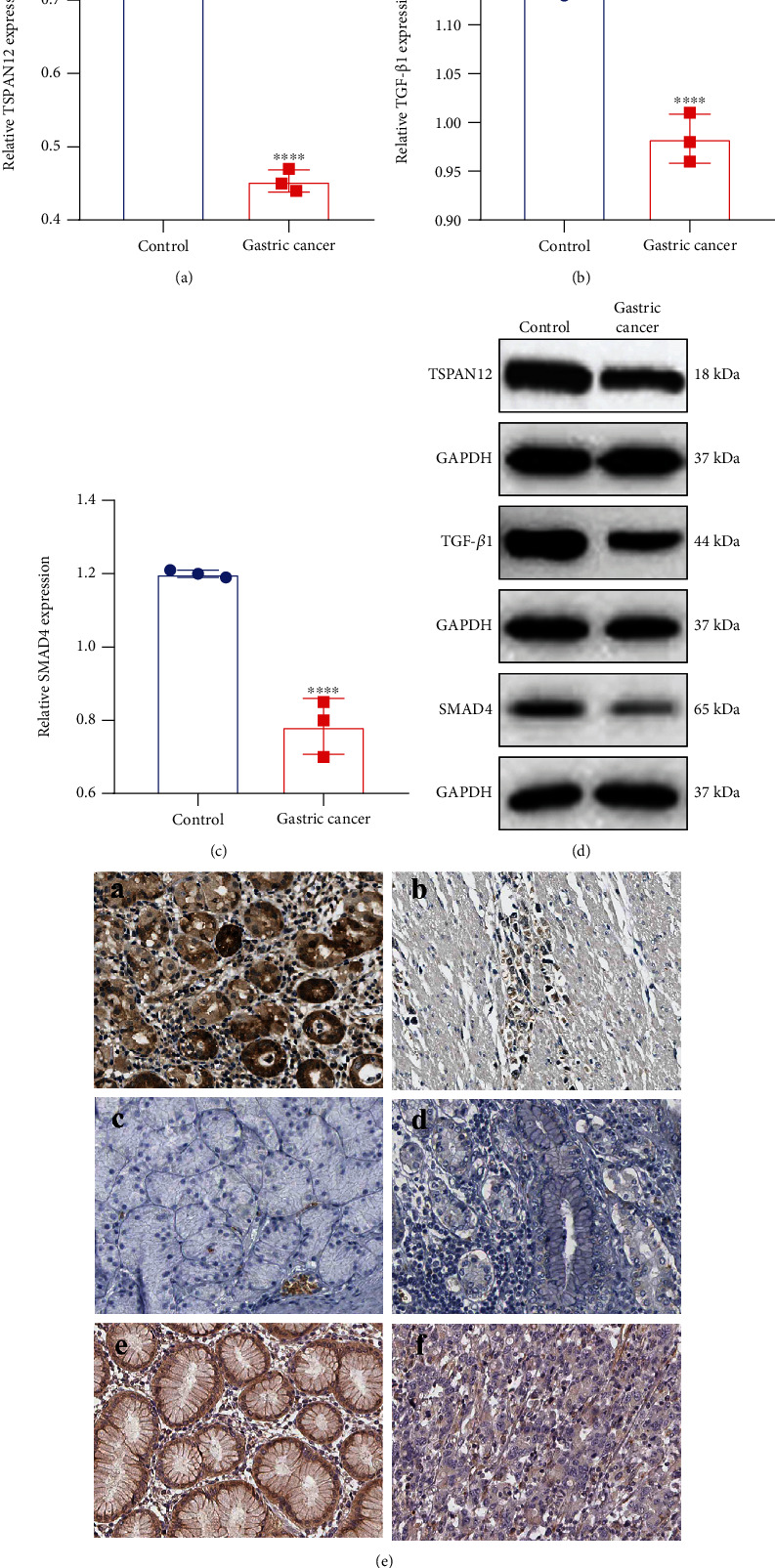
Inactivation of the TSPAN12 expression and TGF-*β*1/SMAD4 pathway in gastric cancer. (a–c) TSPAN12, TGF-*β*1, and SMAD4 expression was tested in gastric cancer and normal tissues utilizing western blot. (d) Representative images of western blots. (e) Immunohistochemistry results of (a, b) TSPAN12, (c, d) TGF-*β*1, and (e, f) SMAD4 in gastric cancer and normal tissues. ^∗∗∗∗^*p* < 0.0001.

Western blot was utilized to detect TSPAN12, TGF-*β*1, and SMAD4 expression in gastric cancer tissues and normal tissues. In Figure 3(a), TSPAN12 expression was distinctly weakened in gastric cancer tissues than normal tissues. Furthermore, low expression of TGF-*β*1 (Figure 3(b)) and SMAD4 (Figures 3(c) and 3(d)) was found in gastric cancer tissues. The above results were confirmed by immunohistochemistry (Figure 3(e)).

### 3.4. FKB Activates the TSPAN12 Expression and TGF-*β*1/SMAD4 Pathway in Gastric Cancer Cells

**Figure 4:**
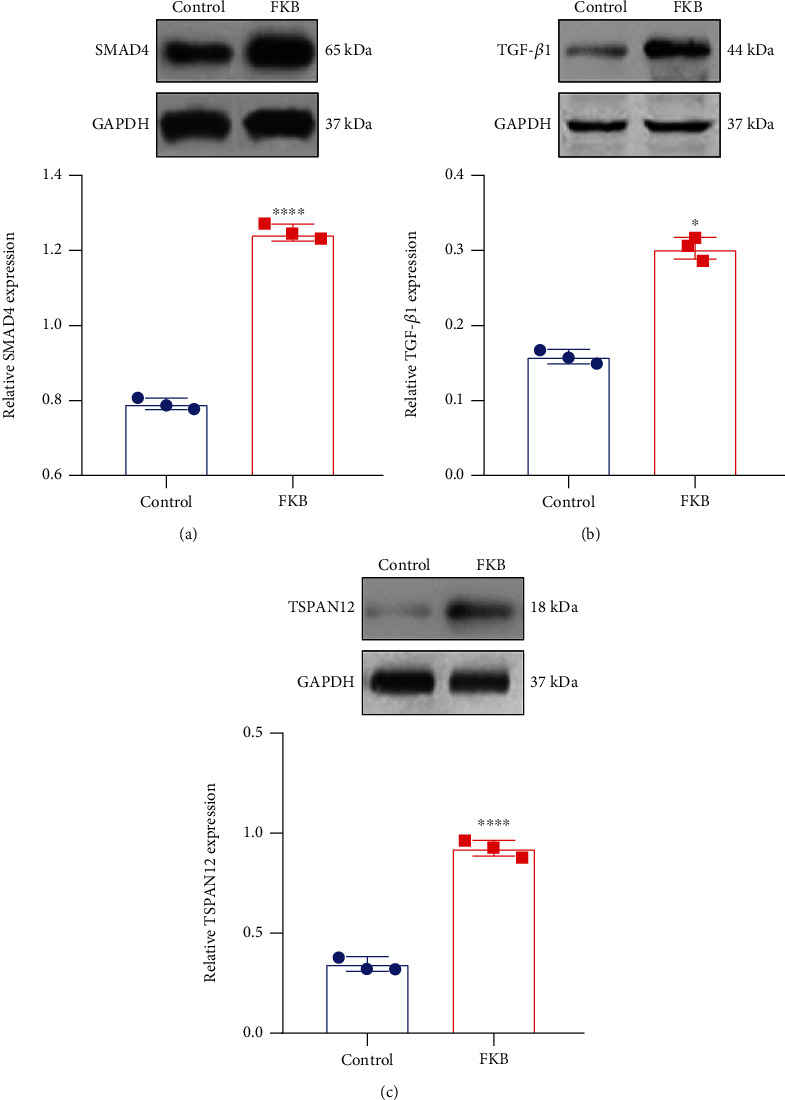
FKB activates the TSPAN12 expression and TGF-*β*1/SMAD4 pathway in gastric cancer cells. (a) SMAD4, (b) TGF-*β*1, and (c) TSPAN12 expression was quantified in SGC-7901 cells treated with FKB. ^∗^*p* < 0.05; ^∗∗∗∗^*p* < 0.0001.

TSPAN12, TGF-*β*1, and SMAD4 proteins were examined in SGC-7901 cells treated with FKB via western blot. FKB treatment prominently increased SMAD4 (Figure 4(a)), TGF-*β*1 (Figure 4(b)), and TSPAN12 (Figure 4(c)) expression in SGC-7901 cells. The results indicated that FKB could exert an anticancer effect via activation of TSPAN12 and the TGF-*β*1/SMAD4 pathway.

### 3.5. FKB Suppresses Proliferation and Accelerates Apoptosis of Gastric Cancer Cells Related to SMAD4

**Figure 5:**
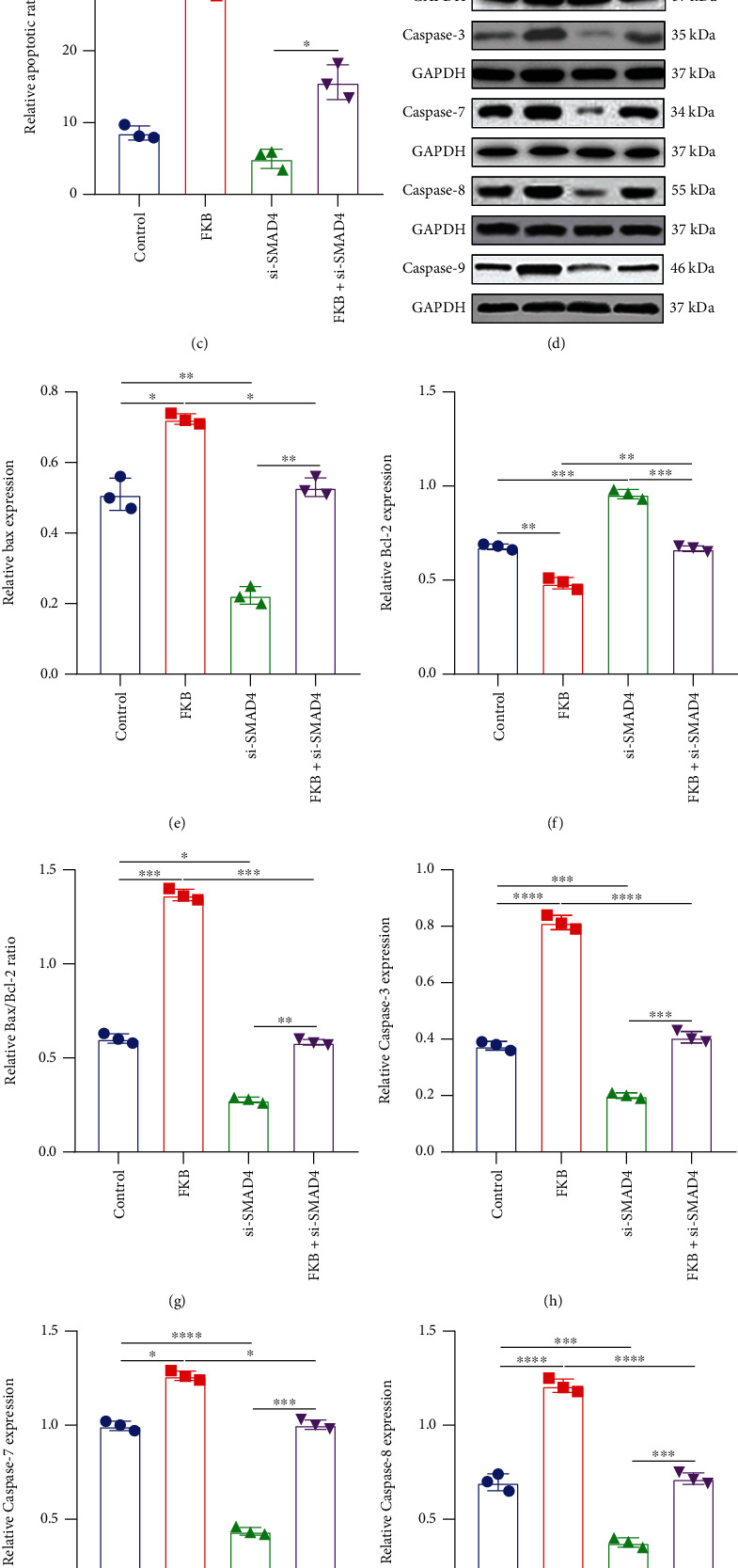
FKB suppresses growth and accelerates apoptosis of gastric cancer cells partly through SMAD4. (a) Cell growth curve for SGC-7901 cells after treatment with FKB and/or si-SMAD4. (b, c) Apoptosis of SGC-7901 cells was investigated when transfection with FKB and/or si-SMAD4 utilizing Annexin V-FITC/PI test. (d) Representative images of western blot. (e) Bax, (f) Bcl-2, (g) Bax/Bcl-2, (h) caspase-3, (i) caspase-7, (j) caspase-8, and (k) caspase-9 expression was quantified in SGC-7901 cells transfected with FKB and/or si-SMAD4. ^∗^*p* < 0.05; ^∗∗^*p* < 0.01; ^∗∗∗^*p* < 0.001; ^∗∗∗∗^*p* < 0.0001.

We next probed into the mechanisms of FKB treatment on proliferative and apoptotic capacities of gastric cancer cells. si-SMAD4 was utilized to silence SMAD4 in SGC-7901 cells. CCK-8 results demonstrated that the cellular proliferative capacities were boosted after transfection with si-SMAD4 (Figure 5(a)). Following cotransfection with FKB and si-SMAD4, the cellular proliferative capacities were suppressed in comparison with the si-SMAD4 group (Figure 5(a)). Compared to controls, the apoptotic rate of SGC-7901 cells was accelerated by FKB treatment, which was inhibited by si-SMAD4 (Figures 5(b) and 5(c)). In comparison with the si-SMAD4 group, the apoptosis rate of SGC-7901 cells was improved when cotransfection with FKB and si-SMAD4. These findings revealed that FKB could suppress growth and accelerate apoptosis of gastric cancer cells, which was related to SMAD4. We also found that SMAD4 knockdown inhibited Bax expression (Figures 5(d) and 5(e)) and promoted Bcl-2 expression (Figure 5(f)) in SGC-7901 cells in comparison to controls. Moreover, the Bax/Bcl − 2 ratio was decreased when transfection with si-SMAD4 (Figure 5(g)). si-SMAD4 transfection decreased the expression of caspase-3, caspase-7, caspase-8, and caspase-9 in SGC-7901 cells (Figures 5(h)–5(k)). However, FKB treatment obviously reversed the expression of above proteins induced by si-SMAD4. Taken together, FKB could increase proapoptotic proteins and decrease antiapoptotic proteins in gastric cancer cells, which was related to SMAD4.

### 3.6. FKB Weakens the Expression of Cell Cycle-Related Proteins and TGF-*β*1 in Gastric Cancer Cells Related to SMAD4

**Figure 6:**
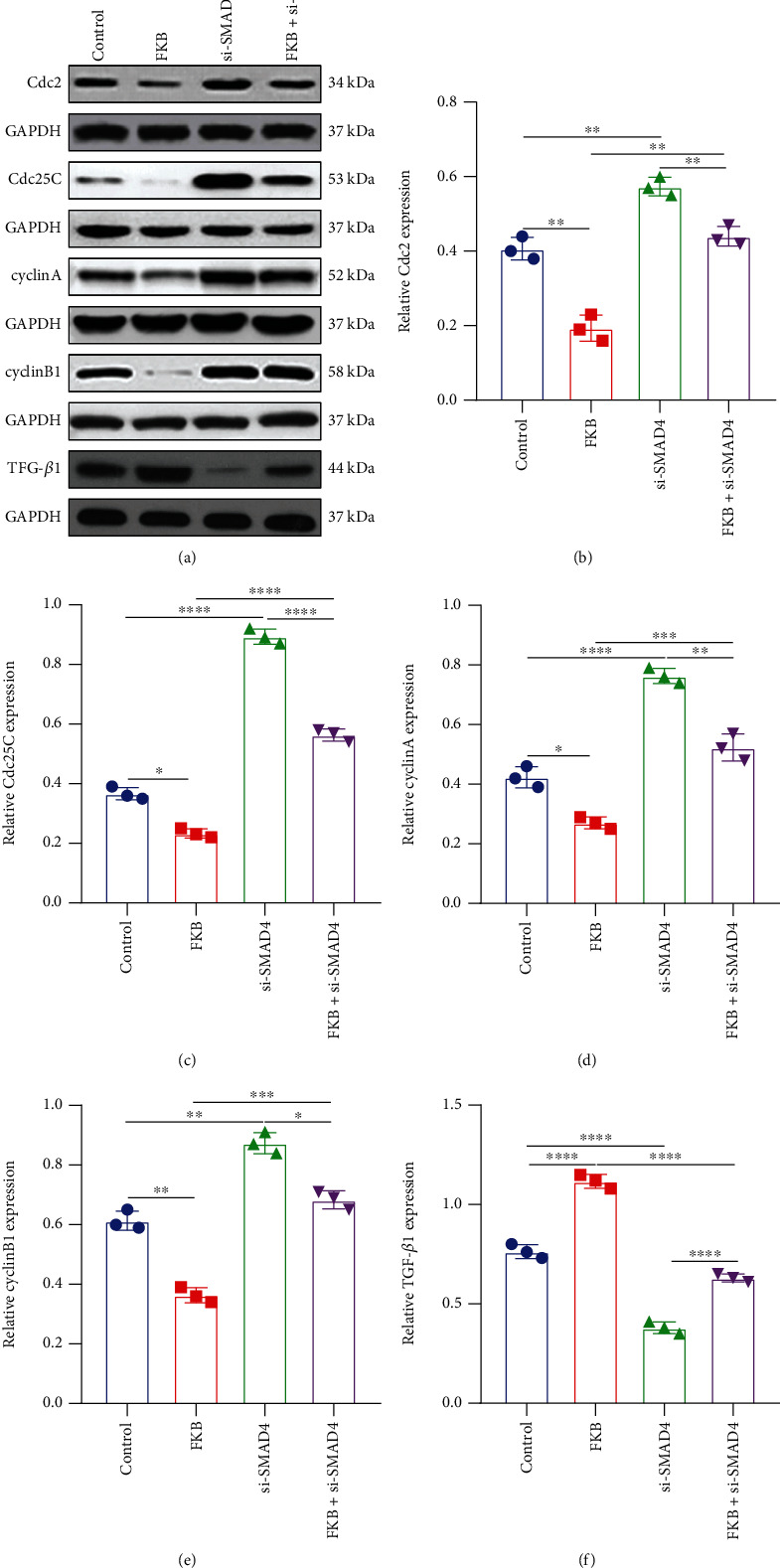
FKB weakens the expression of cell cycle-related proteins and TGF-*β*1 in gastric cancer cells related to SMAD4. (a) Representative images of western blots. (b) Cdc2, (c) Cdc25C, (d) cyclin A, (e) cyclin B1, and (f) TGF-*β*1 expression was quantified in SGC-7901 cells transfected with FKB and/or si-SMAD4. ^∗^*p* < 0.05; ^∗∗^*p* < 0.01; ^∗∗∗^*p* < 0.001; ^∗∗∗∗^*p* < 0.0001.

Western blot was presented to determine the expression of cyclins, CDKs, and TGF-*β*1 in SGC-7901 cells transfected with FKB and/or si-SMAD4 (Figure 6(a)). Firstly, we found that silencing SMAD4 significantly elevated Cdc2 (Figure 6(b)), Cdc25C (Figure 6(c)), cyclin A (Figure 6(d)), and cyclin B1 (Figure 6(e)) expression in SGC-7901 cells compared to controls. However, after cotreatment with FKB and si-SMAD4, the expression of these proteins was distinctly reversed. Furthermore, our data show that TGF-*β*1 expression was markedly decreased by SMAD4 knockdown, which was ameliorated following cotreatment with FKB (Figure 6(f)). The above data suggested that FKB could downregulate cell cycle-related proteins and TGF-*β*1 in gastric cancer cells party through SMAD4.

### 3.7. FKB Activates the TSPAN12 Expression and TGF-*β*1/SMAD4 Pathway In Vivo

**Figure 7:**
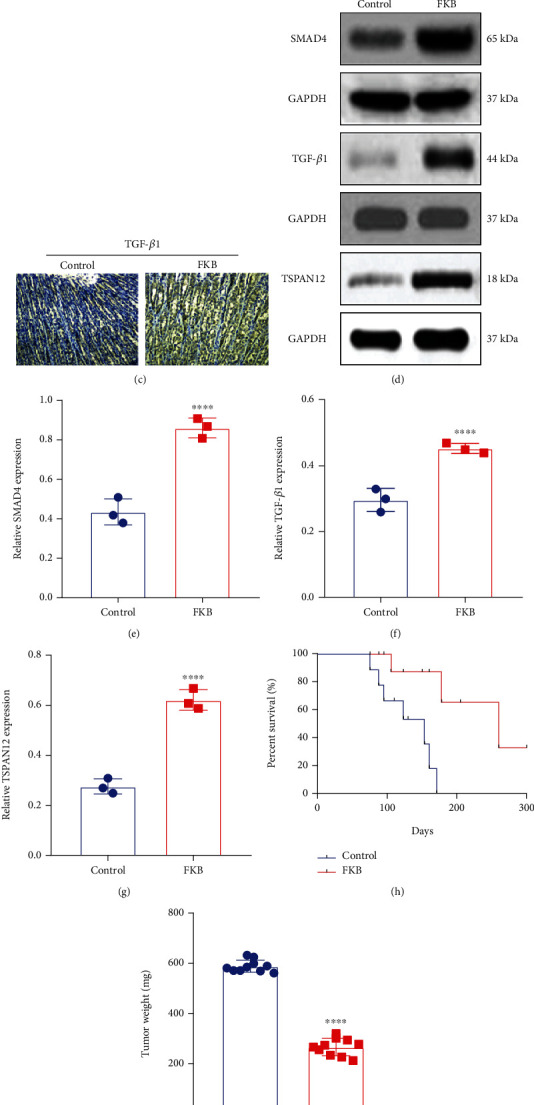
FKB activates TSPAN12 expression and TGF-*β*1/SMAD4 pathway in vivo. (a–c) Immunohistochemistry results of TGF-*β*1, SMAD4, and TSPAN12 proteins in gastric cancer tissues of FKB treatment and control groups. Bar = 20 *μ*m. (d) Representative images of western blot. (e–g) TGF-*β*1, SMAD4, and TSPAN12 expression was examined in gastric cancer tissues of nude mice between FKB treatment and control groups using western blot. (h) FKB treatment prolonged the survival time of nude mice xenografted with SGC-7901 cells. (i) FKB treatment lowered the tumor weight of nude mice xenografted with SGC-7901 cells. ^∗∗∗∗^*p* < 0.0001.

The anticancer effects of 4FKB were assessed using nude mice in vivo. SGC-7901 cells were xenografted into nude mice. There were no signs of toxicity in any of them. We examined TGF-*β*1, SMAD4, and TSPAN12 expression in gastric cancer tissues between the two groups. Both in immunohistochemistry and western blot, SMAD4, TGF-*β*1, and TSPAN12 expression was markedly higher in the FKB group than controls (Figures 7(a)–7(g)). The survival time of nude mice was distinctly prolonged after treatment with FKB in comparison to controls (Figure 7(h)). Additionally, the tumor weight of nude mice was lowered by FKB administration than controls (Figure 7(i)).

### 3.8. FKB-Treated Gastric Cancer Cells Polarize Macrophages toward M1 Phenotype

**Figure 8:**
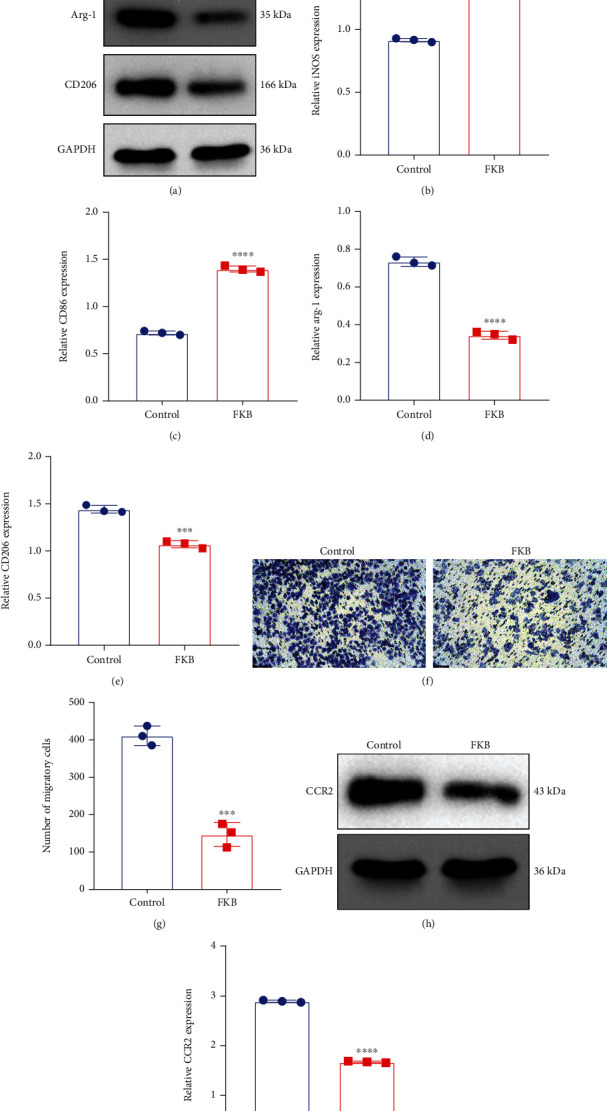
FKB-treated gastric cancer cells polarize macrophages toward M1 phenotype and weaken macrophage migration. (a–e) Western blot of the expression of iNOS and CD86 (M1) as well as Arg-1 and CD206 (M2) in THP-1 cells treated with conditioned media from normal or FKB-treated SGC-7901 cells. (f, g) Transwell of THP-1 cells treated with conditioned media from normal or FKB-treated SGC-7901 cells. Bar, 100 *μ*m. (h, i) Western blot of the expression of CCR2 in THP-1 cells treated with conditioned media from normal or FKB-treated SGC-7901 cells. ^∗∗∗^*p* < 0.001; ^∗∗∗∗^*p* < 0.0001.

Following treatment with 10 *μ*g/mL FKB for 6 h, the cultured media of SGC-7901 cells were exchanged by fresh serum-free media. At 24 h, conditioned media were harvested for treating THP-1 cells. Alterations in THP-1 cell phenotype were evaluated through detecting the surface markers iNOS and CD86 (M1) as well as Arg-1 and CD206 (M2). In comparison to controls, iNOS and CD86 expression was significantly enhanced, and Arg-1 and CD206 expression was significantly decreased for THP-1 cells cultured in conditioned media from FKB-treated SGC-7901 cells (Figures 8(a)–8(e)), indicating that M2 induction capacities were reduced in FKB-treated SGC-7901cells.

### 3.9. FKB-Treated Gastric Cancer Cells Weaken Macrophage Migration

**Figure 9:**
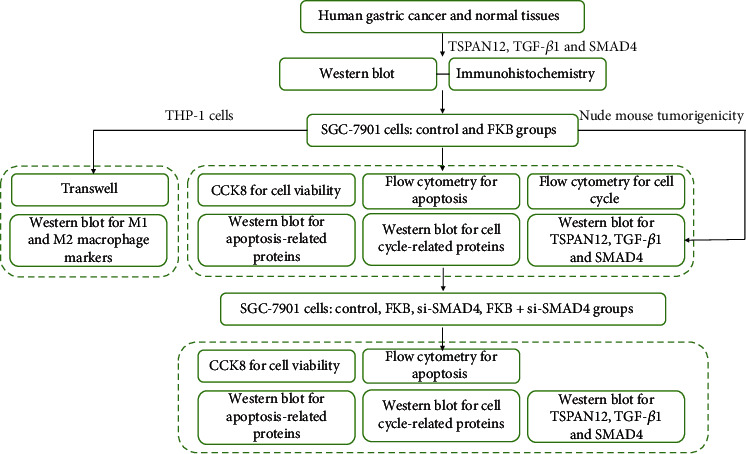
The flowchart of the study design.

Further analysis showed that the migratory capacities of THP-1 cells cultured in conditioned media from FKB-treated SGC-7901 cells were markedly decreased in comparison to that of controls (Figures 8(f) and 8(g)). Additionally, compared with controls, CCR2 expression was significantly reduced in THP-1 cells cultured in conditioned media from FKB-treated SGC-7901 cells (Figures 8(h) and 8(i)). Altogether, FKB-treated gastric cancer cells weakened macrophage migration.  Figure 9 illustrates the flowchart of the study design.

## 4. Discussion

Conventional antitumor drugs for the treatment of gastric cancer, such as doxorubicin and methotrexate, are often limited due to their systemic toxicity and lack of specificity [40, 41]. Therefore, it is necessary to develop effective drugs. Many studies have shown that natural compounds derived from food and plants possess anticancer effects, including FKB [29]. FKB has been fully proven to have great potential as an anticancer agent [29]. Previous research has demonstrated that FKB has cytotoxic effects on human colon cancer (LoVo) [32], lung adenocarcinoma (A-549) [33], prostate cancer (PC3 and DU-145) [29], and squamous cell carcinoma (KB) [42] cells. FKB could induce apoptosis as well as G2/M cell cycle arrest of different cancer cells [32, 43]. In this study, we extracted the protein from gastric cancer tissues and found that the protein expression of TGF-*β*1, SMAD4, and TSPAN12 was prominently decreased in gastric cancer. Intriguingly, FKB treatment significantly increased their expression in gastric cancer cells. For the nude mouse gastric cancer model, the survival time of the FKB treatment group was significantly improved. Furthermore, FKB treatment significantly inhibited cell proliferation as well as accelerated cell apoptosis and G2/M arrest for SGC-7901 cells. These data suggest that FKB could play an antitumor effect related to the TGF-*β*1/SMAD4 pathway.

Apoptosis can balance cell division and cell death. As we all know, apoptosis is related to cancer, and apoptosis inducers have been explored as new ideas for cancer treatment. Herein, we evaluated the apoptosis regulatory mechanism of FKB in gastric cancer cells. Apoptosis is controlled by mitochondrial [44] and cell-surface death receptor pathways [45]. As a previous study, FKB-induced apoptosis depends largely on the damage of mitochondria [42]. The loss of mitochondrial membrane potential induces the release of cytochrome c from the mitochondria into the cytoplasm, which binds to the apoptotic protease activator 1 and caspase-9/7, thereby the activation of the downstream of caspase-3 [30]. In this study, we found that FKB promoted the expression of caspase-3, caspase-7, caspase-8, and caspase-9, suggesting that FKB may accelerate the apoptosis of gastric cancer cells partly through the mitochondrial pathway [46]. Apoptotic Bax and proapoptotic Bcl-2 are involved in the endogenous apoptotic pathway [47, 48]. The proapoptosis activity of Bax protein can be closely related to Bcl-2 protein. Both mediate cell apoptosis through homologous and heterologous complexes. Therefore, the Bax/Bcl − 2 ratio accelerates the execution of apoptosis [49]. In this study, the increase in FKB-induced apoptosis was in association with the decrease in the expression of Bcl-2 and Bax proteins. These results indicate that FKB can interfere with Bax/Bcl − 2, thereby causing apoptosis of gastric cancer cells. Data from current studies indicate that FKB induces apoptosis through the mitochondrial pathway and the cell membrane death receptor pathway.

The destruction of the cell cycle is one of the goals of the development of novel anticancer drugs. A few studies have shown that FKB induces G2/M blockage in leiomyosarcoma (LMS) cells, H460 cells, and ACC-2 cells [50–52]. This cell cycle arrest is related to the reduction of cyclin A, cyclin B1, Cdc2, and Cdc25C. Cdc2 kinase is mainly activated during the G2/M phase, which is in association with cyclin A and cyclin B1 [53]. At the beginning of mitosis, both Cdc2C and cyclin B1 residues are dephosphorylated by Cdc25C [54]. Inactivation of Cdc2 leads to G2/M stagnation after DNA damage [55]. Our data suggest that the inhibition effect of FKB on gastric cancer cell growth may be related to the regulation of cell cycle-related proteins including cyclin A, cyclin B1, Cdc2, and Cdc25C. In a previous study, it has been reported that FKB may induce cell apoptosis by p21-induced cell cycle arrest and activation of p38 in cervical cancer HeLa cells [56]. These findings confirm the antiapoptosis effect of FKB by regulating various cell cycle-related proteins in the treatment of cancers.

As we all know, SMAD4 is the main signal transduction pathway downstream of TGF-*β*. Evidence indicates that SMAD4 can act as a tumor suppressor gene in gastrointestinal cancer [57–59]. TGF-*β* has a potential tumor suppressor effect via inhibiting proliferative and apoptotic abilities of gastric cancer cells [21, 59]. As shown in this study, it was observed the decrease in the expression of TGF-*β*1 and SMAD4 proteins in gastric cancer tissues. This provided potential evidence that downregulated TGF-*β*1 and SMAD4 might participate in gastric cancer progression. TGF-*β*-induced cell cycle arrest is the induction of CDK inhibitor expression [60]. Moreover, TGF-*β* also limits the formation of gastric cancer by activating the apoptotic pathways [21]. From the data we obtained, FKB can induce the increase in the expression of TSPAN12 protein, suggesting that TSPAN12 may be a target for the treatment of FKB. Our data showed that after transfection with si-SMAD4, the therapeutic effect of FKB was significantly weakened, indicating that FKB may inhibit gastric cancer partly through the TGF-*β*1/SMAD4 pathway. Also, FKB treatment prolonged the survival time of nude mice. We also demonstrated iNOS and CD86 expression was significantly enhanced, and Arg-1 and CD206 expression was significantly decreased in THP-1 cells cultured in conditioned media from FKB-treated SGC-7901 cells. This demonstrated that FKB enabled to weaken the capacity of gastric cancer cells in inducing M2 macrophage polarization. Additionally, FKB-treated SGC-7901 cells weakened macrophage migration. Therefore, FKB may be a promising antigastric cancer drug, but its effect and mechanism deserve more in-depth study.

## 5. Conclusion

Taken together, our findings confirmed the antigastric cancer effect of FKB both in gastric cancer cells and nude mice with gastric cancer. Mechanically, FKB treatment could exert antiproliferation and proapoptosis effects, which was related to the TGF-*β*1/SMAD4 pathway. Additionally, FKB treatment weakened M2 macrophage polarization induction capacities of gastric cancer cells.

## Data Availability

The datasets analyzed during the current study are available from the corresponding authors on reasonable request.

## Abbreviations

FKB: Flavokawain B
CDKs: Cyclin-dependent protein kinases
TGF: Transforming growth factor
